# Medical Opinion and Movement

**Published:** 1911-09-16

**Authors:** 


### Medical Opinion and Movement.

### Constipation and Epilepsy.

Constipation has frequently been cited as a contributory causative factor in the production of epileptic fits, but Dr. Axtell, of Washington, claims to have discovered a condition of "acute angulation and flexure of the sigmoid" as a common cause of epilepsy. During the last two years he has found the condition present in ten private and twenty-six asylum cases of epilepsy, and since the condition has been sought for it has only been absent in one case admitted to the asylum, and in that case the epilepsy was produced by a depressed fracture. Of course the author admits that the condition may be present without producing epilepsy. Moreover it will only be found as a causative factor in those cases where the epilepsy has been acquired in later years, when irregular habits of living have been more or less fairly established. The author reports full details of several cases of this nature in the *American Journal of Surgery*. In all of them there was a history of persistent obstinate constipation, with abdominal distension, flatulence, and distress. The condition can only be clearly established by examination with the sigmoidoscope after thorough clearing of the bowel by means of enemata. In all the cases so treated, the epileptic condition was improved by treatment of the bowel condition, but only three of the patients persisted in the treatment sufficiently long to be quite freed from the epileptic trouble. In some cases active treatment actually increased at first the frequency of the convulsions, a fact which, according to the author, is convincing evidence of the close connection between the two pathological conditions.

### Scarlatina and Cutaneous Hæmorrhages.

Some few years ago, Dr. Hecht drew attention to the fact that in a patient suffering from scarlet fever, if a fold of skin on the back or chest were raised and compressed between the finger and thumb for five to ten seconds, petechial hæmorrhages were produced, and he considered that in cases in which these hæmorrhages were not easily produced in this way, the diagnosis of scarlet fever was doubtful. Dr. C. Leede, of the Allgemeine Krakenhaus of Hamburg-Eppendorf, has since formed a similar conclusion without knowledge of Dr. Hecht's work, and gives the results of his observations on two hundred cases in the *Münchener Medizinische Wochenschrift*. He noticed in the course of a severe epidemic of scarlet fever that in carrying out a bleeding, the constricting bandage applied previously to the arm caused the formation of petechiæ, and in some cases quite a hæmorrhagic suffusion, a phenomenon which was not observed in other than scarlatinal cases. Dr. Leede then undertook regular observations on all scarlatinal cases by applying a rubber bandage to the arm sufficiently tight to cause a dilatation of the veins, but without stopping the arterial pulse. After ten to fifteen minutes the bandage was removed and, by stretching the fold of skin at the elbow, the petechiæ were easily shown. According to the author the phenomenon never fails in cases of scarlet fever, but it may also be present, though to a less extent, in measles. This weakness of the capillary walls diminishes during convalescence, so that after twenty-one days it may be entirely absent; but in complicated cases, with nephritis, otitis media, etc., it may be present up to forty-two days. It disappears more rapidly in females than in males.

### Uræmia.

An interesting contribution to the discussion of this subject by Drs. Obermayer and Popper appears in the *Zeitschrift für Klinische Medizin*. They accept the general supposition that uræmia is an intoxication produced by the retention in the blood of urinary constituents, or by the insufficient elimination of the products of organic metabolism. They suggest, however, that the variations in the clinical manifestations of uræmia and the differences in the power of elimination of the kidneys from one case to another point to the probability of a multiplicity of poisons in the causation of the uræmic condition. According to their analyses, however, of a large number of cases, indican is almost invariably present in the blood-serum in cases of uræmia, whereas in the absence of uræmia they do not find it present in the healthy subject nor in patients suffering from a variety of diseases—pneumonia, septicæmia, hepatic or cerebral affections, cardiac disease, etc. According to these authors, therefore, indicanæmia may be considered characteristic of a uræmic condition and has considerable diagnostic significance. Although well-marked uræmic symptoms are unmistakable, initial uræmic phenomena, especially of a cerebral nature, are not infrequently confused with cerebral affections, and in such cases the evidence of indican in the blood would point to the source of the trouble in the kidneys. According to the authors, indicanæmia has also a prognostic significance. Of twenty-four patients in whom indican was found present in the blood, twenty-three died within a short time. In the twenty-fourth case the condition improved for a while, but the patient succumbed a few weeks later.

### The Preliminary Perineal Stitch.

For some years Dr. Lapthorn Smith, of Montreal, has practised a plan of placing a perineal suture (or two) in position before the perineum is lacerated. In the *Medical Record* he publishes once more the technique of and argument for this proceeding. Just before the head comes down on to the perineum he anæsthetises the patient and sterilises the perineum with antiseptics. He then places the left thumb in the rectum and the left forefinger in the vagina, and enters a perineum needle at the lower end of the labium minus on the (patient's) left side. Taking in the levator ani, and passing under the vagina about two and a half inches above the fourchette, the needle is made to emerge at a corresponding place on the right side. A piece of silkworm gut is then threaded to the needle and the latter withdrawn. A second needle is then passed an inch lower down, taking in the muscles of the perineum. Should there ensue no laceration the stitches are withdrawn; otherwise they are tied from above downwards immediately after delivery is complete. The author says this method does not interfere in any way with the normal course of labour nor with the use of the forceps. One of his points in favour of this preliminary stitching is that there is no trouble about securing the divided and retracted ends of the levator ani muscle, even for the least expert.

### Action of Animal Extracts on the Bladder.

Some experiments carried out by Drs. Ott and Scott in Philadelphia have convinced the former that he was wrong some time ago when he attributed the action of pituitary extract on the bladder to the vesicospinal centre and not to the unstriped muscle. Though still maintaining that there is some effect on the centre, they now concede that the main action is directly on the muscle fibres. They have also examined the action of other animal extracts, and have published in the *Medical Bulletin* a list of the experience gained. The solutions were injected into the jugular vein of etherised cats. Thyroid extract produces strong contraction of the bladder (when the pelvic nerves are intact). Prostatic extract increases both the force and frequency of the vesical contractions. Parathyroid increases the tone of the bladder wall, whereas adrenalin lowers it. Mammary gland has no action at all. Pancreas and parotid have considerable action in increasing the contractions; orchitic extract and thymus do the same, but in a lesser degree; and brain and ovary have only slight action, which are of the same nature. It is curious to notice that nearly all these extracts have similar actions upon the unstriped bladder muscle, with the single exception of adrenalin.

### A New Routine for Gonorrhœa.

In the *International Journal of Surgery* Dr. Geis advocates strongly a system of treatment for gonorrhœa in the female which he has been trying for seven years. He emphasises the importance of entire rest (when patients can and will take it), the danger of using powerful antiseptics, and the necessity of free purgation. He also gives urotropin or salol by the mouth, with copious draughts of water, and believes that this alone will cure some cases. When the brunt of the infection falls upon the vagina he relies upon regular douching with a hot saturated solution of picric acid in equal parts of water and glycerine. (The glycerine must be added to the aqueous solution, not *vice versa*.) Four quarts of this are to be used, with the patient on her back, with raised pelvis. When, however, the infection especially affects the external genitalia, the author paints both surfaces of both labia, the clitoris, vestibule, urethral meatus, vaginal opening, and perineum with a fifty per cent. solution of argyrol in glycerine. The douche is used every four hours, and gauze soaked in picric acid solution is applied to the vulva in between. If the cervix is also involved he paints the surface of it with the same argyrol solution, and leaves in a tampon soaked in picric acid glycerine.

### Diphtheritic Palsy.

The central as opposed to the peripheral origin of diphtheritic paralysis is maintained in the *Long Island Medical Journal* by Dr. Spingarn. This author holds that the currently accepted view of this condition as a peripheral neuritis is wrong, and is based upon clinical evidence mainly rather than on exact research. The failure to demonstrate central lesions in fatal cases is a failure, he believes, of technique, and he draws a close parallel between the nerve lesions of the diphtheritic toxin and those of the virus of epidemic poliomyelitis. In support of this theory he quotes many observations by Continental observers, and especially the well-known loss of knee-jerk so often seen early in diphtheria cases, and the Babinski reflex recently noted by Dr. J. D. Rolleston. Both of these must be attributed to a central lesion, for no peripheral lesion could have loss of knee-jerk for its sole manifestation. He then reports two fatal cases of diphtheritic paralysis from his own practice, and puts forward the view that the cause of death was a vagus degeneration rather than a direct cardiac effect. The point is argued with ability, but would be more convincing if microscopic and other evidence were adduced in favour of it. Dr. Spingarn's evidence is as purely clinical as that upon which the peripheral theory is believed in, and he has a lot of work to do before he can expect much support for his hypothesis.

### The Deaf Child.

Under this title Dr. Kerr Love, of Glasgow, has published a small book (to be obtained from Messrs. Wright and Sons, of Bristol, 4s. 6d. net) which gives the results of his investigations into the conditions under which deaf children are taught in this country, on the Continent, and in America. The education of the deaf is a matter which should interest every practitioner, but it is a lamentable fact that few men in general practice are fully aware of the amount of harm that is likely to be done by adherence to the older methods of training, of fully cognisant of the possibilities of the oral method. Specially worthy of attention are the chapters dealing with the examination of deaf children—the diagnosis of deafness, in fact—and with the treatment of deaf children generally. The author lays, perhaps, too little stress on the possibilities of surgical interference, but he rightly urges that the first indication for the treatment of deafness is the removal of post-nasal adenoids and enlarged tonsils, if these are present. Yet, as he remarks, even with school clinics and better surgical treatment, the real problem lies in the education of the deaf child, and to the consideration of this important aspect of the subject the main portion of his book is devoted. To teachers no less than to practitioners Dr. Love's little monograph should prove of instructive interest.

### The Crawl Treatment of Scoliosis.

Some time ago we pointed out, in a special article dealing with Professor Klapp's clinic at Berlin ("The Klapp Treatment of Scoliosis," The Hospital, January 14, 1911, pp. 463-65), the importance of this method of dealing with cases of double curvature of the spine. We are glad to see that Mr. Kellett Smith, in his little book on "Lateral Curvature of the Spine and Flat Foot," published recently by Wright and Sons, of Bristol, recognises the importance of the method, and describes it carefully, with indications for its application. Mr. Smith's essay is a valuable contribution to the study of scoliosis, especially as regards its treatment. To orthopædists, indeed, there is little in it that is original or novel, but as a summary of the modern methods of diagnosis and treatment it is excellent, and as such worth buying by the practitioner who desires to have at hand a useful manual on a much-neglected subject. Admirably illustrated by means of photographs, showing various curves and the exercises employed in the Klapp method, it is a really helpful manual for those who have not the time or opportunity to refer to larger works. We must confess, however, that we are not so enthusiastic concerning that part of the book which deals with flat foot. The author has not found, he declares, much benefit result from the wearing of pads, but he does not describe the way in which these pads are manufactured, and it seems to us that the reason for his dissatisfaction is to be found in the fact that he used varieties of the pads "on the market." The only useful pad is one manufactured by the orthopædist himself, or at least under his immediate supervision, on an actual corrected cast of the patient's foot. With a celluloid pot, a piece of flat cork, and a little care and trouble, such pads can easily be made, and in general they are far superior to the costly and useless articles dispensed by the instrument makers. Manipulative exercises of the foot, which are fully described, are excellent, but we have little faith in the walking and toe exercises recommended in the treatment of flat foot. A valuable and instructive chapter at the end is devoted to tonic treatment, and here the only suggestion we have to make is that the author should in a second edition point out that one of the cheapest and most efficient substitutes for cod-liver oil is cotton-seed oil, which, as the mistura ol. gossypii, is extensively used in some hospitals. It is readily assimilated, and is better than the inferior grades of olive oil now so largely employed.

### X-Rays in Hyperidrosis.

The *X*-rays have sometimes been found most successful in the treatment of hyperidrosis. The armpit is one of the parts in which excessive sweating is occasionally a source of extreme discomfort and annoyance to persons who are otherwise in good health. The patient may be either a man or a woman. Up till breakfast time all may be well, and then immediately afterwards the local perspiration may set in so actively that the clothes in the region of the axillæ have to be changed, and possibly more than once in the day. Dr. Howard Pirie has recently pointed out the value of the *X*-rays in the relief of this condition. He has also pointed out how there are two classes of cases, one in which, as might be expected, the perspiration is chiefly upon hot days, the other in which, contrary to expectation, local sweating is increased by cold. Measured doses of *X*-rays were applied once a month for four months, at the end of which time every case had either ceased to perspire noticeably at all or else the amount of perspiration was at least reduced to less than normal. Some of the patients were medical men.

### Scarlet Red in Accelerating the Skinning Over of Granulating Surfaces.

The difficulty often met with in causing the central parts of large burns, bed-sores, or ulcers to skin over is familiar enough, and the necessity for skin-grafting in many of these cases is obvious; but even with skin-grafting it sometimes happens that the re-covering of granulating surfaces with stratified epithelium is either very slow or does not take place at all. Any dressing which may assist the process will be welcomed, and amongst the newer applications may be mentioned in particular scarlet red and its component amido-azo-toluol, which has been experimented with largely both on the Continent and in America. It is generally applied in the form of an ointment of the strength of about 8 per cent., when the granulating surface has been rendered clean by the use of boric acid or other mild antiseptic. It is as well to protect the skin around the granulating surface before applying the scarlet red ointment, in order to prevent irritation of that part which has already become epithelialised. The 8 per cent. scarlet red ointment can then be applied after being previously smeared upon lint, and as a rule it is best to alternate its use with an application of bland ointment each twenty-four or forty-eight hours. Some prefer to start with a 4 per cent. ointment, in which case the danger of undue irritation is minimised, but, especially if the ulcerated surface has already been long stationary before the scarlet red has to be employed, it may easily happen that a 4 per cent. ointment is insufficient to stimulate the epithelial cells, so that it may fail to accomplish the object in view. Roughly speaking, therefore, an 8 per cent. ointment, alternating every one or two days with a bland ointment, is that which should be used as a routine procedure in these cases. This ointment is now being extensively used with the best results in some London hospitals.

